# Ferroptosis-Based Nanotherapeutic Strategies to Overcome Temozolomide Resistance in Glioblastoma: A Systematic Review and Meta-Analysis

**DOI:** 10.3390/curroncol33040194

**Published:** 2026-03-30

**Authors:** Yashaswi Sharma, Arpana Parihar, Neha Arya, Jagat Kanwar, Murali Munisamy, Megha Katare-Pandey, Ashwani Tandon, Mahadev Rao, Saikat Das, Adesh Shrivastava, Rashmi Chowdhary, Amit Agrawal, Rupinder Kaur Kanwar

**Affiliations:** 1 Department of Translational Medicine, All India Institute of Medical Sciences, Saket Nagar, Bhopal 462020, MP, India; yashaswi.phd2024@aiimsbhopal.edu.in (Y.S.); arpana.postdoc2024@aiimsbhopal.edu.in (A.P.); neha.tmc@aiimsbhopal.edu.in (N.A.); murali.tmc@aiimsbhopal.edu.in (M.M.); megha.tmc@aiimsbhopal.edu.in (M.K.-P.); 2 Department of Biochemistry, All India Institute of Medical Sciences, Saket Nagar, Bhopal 462020, MP, India; jagat.biochemistry@aiimsbhopal.edu.in (J.K.); rashmi.biochemistry@aiimsbhopal.edu.in (R.C.); 3 Department of Pathology, All India Institute of Medical Sciences, Saket Nagar, Bhopal 462020, MP, India; ashwani.patho@aiimsbhopal.edu.in; 4 Department of Pharmacy Practice, Manipal College of Pharmaceutical Sciences, Manipal Academy of Higher Education, Manipal, Udupi 567104, KA, India; mahadev.rao@manipal.edu; 5 Department of Radiation Oncology, All India Institute of Medical Sciences, Saket Nagar, Bhopal 462020, MP, India; saikat.radiotherapy@aiimsbhopal.edu.in; 6 Department of Neurosurgery, All India Institute of Medical Sciences, Saket Nagar, Bhopal 462020, MP, India; adesh.neurosurgery@aiimsbhopal.edu.in

**Keywords:** temozolomide resistance, glioblastoma, ferroptosis, nanoparticle, therapeutics

## Abstract

Glioblastoma multiforme (GBM) is one of the most aggressive and treatment-resistant forms of brain cancer, posing challenges to modern oncology. Current treatments, including surgery, radiation, and chemotherapy (e.g., Temozolomide or TMZ), often fail due to the inevitable development of drug resistance. TMZ resistance remains a major therapeutic challenge for the reasons that it is the first-line treatment. Recent studies indicate a rising GBM tumour burden and a trend towards earlier age of onset. It highlights the urgent need for evidence-based policymaking and intensified research to address this most difficult-to-treat malignancy in clinical settings. Ferroptosis, a newly recognized type of controlled cell death induced by iron-dependent lipid peroxidation, has emerged as a potential approach to overcome apoptosis resistance and restore drug sensitivity in GBM. This mechanism is modulated by key molecules that can be specifically targeted to either enhance oxidative stress or inhibit antioxidant defences, ultimately leading to tumour cell death. This review conducts a meta-analysis of preclinical evidence to better understand the potential of activating ferroptosis as a key target for developing nanoparticles to resensitize TMZ-resistant GBM cells. Current evidence indicates that combining ferroptosis induction with strategically engineered nanocarrier systems can serve as a novel and effective therapeutic approach to overcome TMZ resistance and advance precision-based GBM treatment.

## Simple Summary

Glioblastoma multiforme (GBM) is one of the most aggressive and treatment-resistant forms of brain cancer, posing challenges to modern oncology. Despite advancements in the current standard of care, which includes radiotherapy, surgery, and medication (usually chemotherapy with concurrent temozolomide (TMZ)), the median overall survival (OS) for patients is still between 14.6 and 20.5 months, making the outcome nearly always fatal. Current treatments, including surgery, radiation, and chemotherapy (e.g., TMZ), often fail due to the inevitable development of drug resistance. TMZ resistance remains a major therapeutic challenge for the reasons that it is the first-line treatment. Drug combinations that target ferroptosis signalling enhance the efficacy of chemotherapy, radiation therapy, and immunotherapy, as ferroptosis modulates their effectiveness. Combining medications that target ferroptosis pathways is, therefore, a novel therapeutic approach. Current evidence indicates that combining ferroptosis induction with strategically engineered nanocarrier systems can serve as a novel and effective therapeutic approach to overcome TMZ resistance and advance precision-based GBM treatment.

## 1. Introduction

Glioblastoma (GBM) is the most aggressive form of primary brain tumour, originating from astrocytes, the star-shaped glial cells that support nerve cells in the central nervous system. According to the 2021 World Health Organization (WHO) Classification of Tumors of the Central Nervous System, glioblastoma is defined as glioblastoma, IDH-wildtype, CNS WHO grade 4, reflecting advances in molecular diagnostics that distinguish IDH-wildtype glioblastoma from astrocytoma, IDH-mutant, CNS WHO grade 4. This molecular classification has replaced the older terminology that broadly categorized glioblastoma as a “Grade IV glioma”, emphasizing the importance of IDH mutation status in determining tumour biology and clinical behaviour [1]. GBM is characterized by rapid proliferation, diffuse infiltration into surrounding brain tissue, and a high degree of genetic heterogeneity [1,2]. It is a highly malignant brain tumour, accounting for approximately 15% of all central nervous system tumours and roughly 45% of primary malignant brain tumours [3]. Originally, GBMs were thought to arise exclusively from glial cells, but evidence indicates they may arise from a variety of cell types that share characteristics with neural stem cells. Since these cells undergo multiple stages of differentiation from stem cell to neuron to glia, phenotypic variation is mostly driven by molecular changes in signalling pathways rather than by differences in the cell type that originated [1]. GBMs can be classified as primary (de novo), arising without a known precursor, or secondary, in which a low-grade tumour gradually transforms into GBM. From a molecular perspective, this historical distinction closely corresponds to IDH mutation status. Primary glioblastomas are typically IDH-wildtype, whereas tumours historically described as secondary GBMs frequently harbour IDH mutations and are now classified as astrocytoma, IDH-mutant, CNS WHO grade 4 in the WHO 2021 framework. These molecular subtypes exhibit distinct genetic landscapes, metabolic profiles, and therapeutic responses, which may influence mechanisms of temozolomide resistance and susceptibility to ferroptosis-based therapeutic strategies [1,4]. A majority of GBMs are primary, and these patients tend to be older and have a poorer prognosis than patients with secondary GBMs [5]. GBMs can occur at any age, even in children; however, they typically present at a median age of 64. Males have a slightly higher incidence than females (1.6:1) [1,4]. In most patients, no known causes of GBM have been identified. The only known external environmental factor that causes gliomas is high levels of ionizing radiation exposure [6,7]. Despite advancements in the current standard of care, which includes radiotherapy, surgery, and medication (usually chemotherapy with concurrent TMZ), the median overall survival (OS) for patients is still between 14.6 and 20.5 months, making the outcome nearly always fatal [8]. Elderly patients have a significantly worse prognosis; their average survival time after diagnosis is less than 8.5 months. Therefore, new therapeutic approaches are vital for GBM, as the current approved treatments have poor survival rates [8]. Currently, TMZ is the only available first-line chemotherapeutic drug approved for GBM treatment due to its ability to cross the blood–brain barrier (BBB). However, resistance to the drug inevitably develops in most patients, leading to treatment failure. Restoring or enhancing TMZ’s effectiveness is therefore a critically important area of research [9]. Many studies have explored ferroptosis as a viable anticancer approach that can be targeted to overcome treatment resistance [10,11,12,13]. In contrast to necroptosis, pyroptosis, and apoptosis, ferroptosis is a novel form of programmed cell death triggered by iron-dependent phospholipid peroxidation and ultimately leading to cellular death [14,15]. Reactive oxygen species (ROS) buildup and the inactivation of the intracellular reduction system appear to be ubiquitous, in contrast to other forms of apoptotic and non-apoptotic cell death [16]. Changes in cell morphology include blistering and rupture of the cell membrane, contraction and increase in membrane density, loss or reduction in the mitochondrial ridge, rupture of the outer membrane of the mitochondria, and a normal-sized nucleus free of chromatin agglutination [17]. The increase in ROS during iron-dependent redox reactions leads to a redox imbalance in cells, which, in turn, induces ferroptosis. Ferroptosis inducers (FINs) can affect tumour cells that rely on iron for survival [17]. Studies revealed that the primary cause of cancer and the maintenance of tumour malignancy is an iron-rich microenvironment, highlighting the significance of ferroptosis in malignant tumours [18]. Drug combinations that target ferroptosis signalling enhance the efficacy of chemotherapy, radiation therapy, and immunotherapy, as ferroptosis modulates their effectiveness. Combining medications that target ferroptosis pathways is, therefore, a novel therapeutic approach [17]. Additionally, new research has shown that ferroptosis is a viable anticancer approach for overcoming [10,11] treatment resistance [10,11] and can be combined with a variety of anticancer treatments, some of which have received FDA approval [11,12,13]. Additionally, nanoparticles (with a size between 1 and 100 nm) can be combined to develop nanomaterials that facilitate the delivery of anticancer drugs across the BBB and effectively release them at the target site, i.e., the tumour tissue itself [2,19,20,21]. This review conducted a meta-analysis and systematic literature search to better understand preclinical studies investigating ferroptosis induction as a strategy to overcome TMZ resistance in GBM, exploring nanoparticle-based delivery systems to reverse TMZ resistance in GBM, and examining the combined role of ferroptosis and nanoparticle interventions to overcome TMZ-resistant GBM models.

## 2. Materials and Methods

This systematic review and meta-analysis were conducted in accordance with the Preferred Reporting Items for Systematic Reviews and Meta-Analyses (PRISMA) guidelines, and the study protocol was prospectively registered in the PROSPERO database (Registration ID: CRD420251244962). Electronic databases including PubMed, SCOPUS, COCHRANE, and ScienceDirect were searched with the terms outlined in Table 1.

The primary objective was to synthetically evaluate preclinical evidence on ferroptosis induction and nanoparticle-based delivery strategies for overcoming TMZ resistance in GBM. Specifically, the review aimed to (i) systematically assess preclinical studies investigating ferroptosis as a therapeutic mechanism to reverse TMZ resistance, (ii) synthesize evidence on nanoparticle-mediated drug delivery systems designed to enhance TMZ sensitivity, and (iii) evaluate the combined effect of ferroptosis-targeting and nanotechnology-based interventions in sensitizing TMZ-resistant GBM models (Table 2).

Eligible studies included preclinical in vitro or in vivo GBM models with documented TMZ resistance that investigated interventions targeting ferroptosis pathways and/or nanoparticle-based delivery approaches and reported quantitative outcomes such as IC_50_ values, TMZ sensitivity, tumour growth inhibition, or survival. Only full-text articles published in English were considered. Clinical trials, case reports, retracted articles and other studies involving human subjects were excluded, as were diagnostic, formulation, or biomarker studies without therapeutic intervention, studies not addressing TMZ resistance or lacking IC_50_ or treatment-response outcomes, and reviews, editorials, or conference abstracts without complete data. The methodological quality and risk of bias of the included studies were independently evaluated by two reviewers (Y.S. and A.P.). For in vivo animal experiments, the SYRCLE’s Risk of Bias (RoB) tool [22] was employed. Any discrepancies between reviewers were resolved through discussion and consensus with a third author (AA).

### Statistical Analysis

Meta-analysis was conducted using R software (version 4.4.3) with the meta and metafor packages. The primary outcome was measured as the Standardized Mean Difference (SMD), calculated using Hedges’ g, a random-effects model (DerSimonian-Laird) was applied to calculate pooled effect sizes, assuming inherent biological variability between different GBM models and nanoparticle formulations. Heterogeneity was quantified using the I^2^ statistic, where I^2^ > 50% indicated moderate-to-high heterogeneity. Publication bias was assessed with funnel plots and Egger’s linear regression test. All statistical tests were two-tailed, and a *p*-value < 0.05 was considered the threshold for statistical significance.

## 3. Results

### 3.1. Study Characteristics

**Figure 1:**
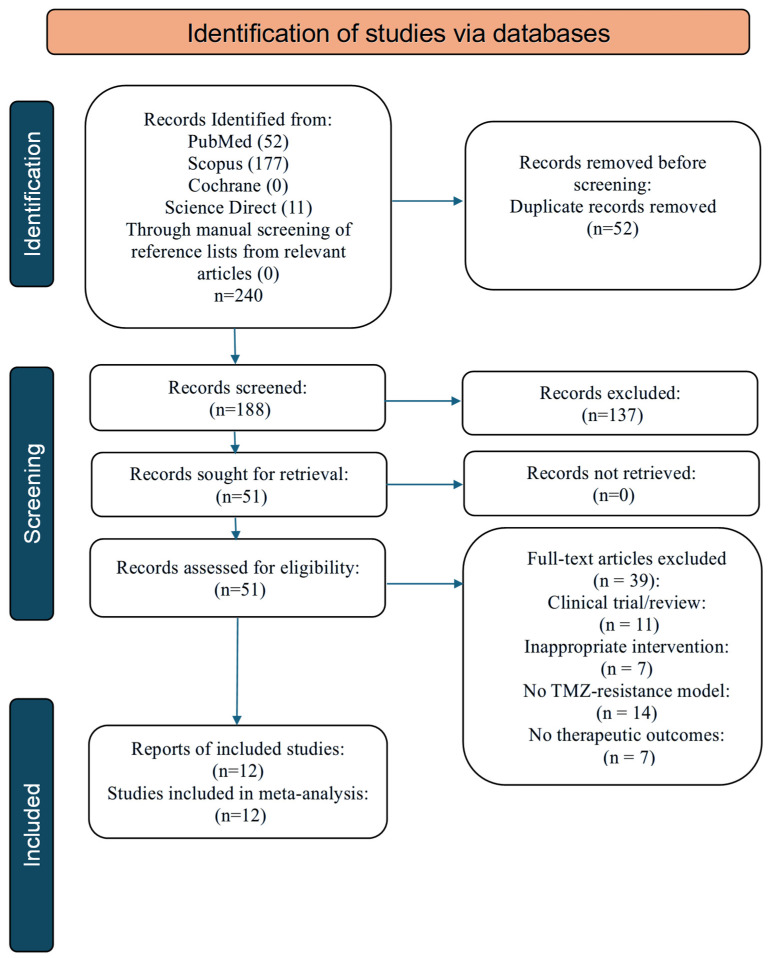
PRISMA flow diagram.

The database search yielded a total of 240 records (Figure 1). After removal of duplicates, 188 records were screened based on titles and abstracts. Of these, 51 full-text articles were assessed for eligibility. Following full-text evaluation, 39 studies were excluded for predefined reasons (Table 3) [23,24,25,26,27,28,29,30,31,32,33,34,35,36,37,38,39,40,41,42,43,44,45,46,47,48,49,50,51,52,53,54,55,56,57,58,59,60,61], resulting in 12 studies being included in the qualitative synthesis (Table 4) [3,62,63,64,65,66,67,68,69,70,71,72].

### 3.2. Molecular and Functional Differences Among GBM Cell Models

The included studies have utilized various GBM models, including established cell lines (T98G, U87, LN18, LN229), GSCs, and patient-derived systems, each characterized by distinct molecular and metabolic profiles [4,5]. These models differ substantially in genetic background, redox regulation, and iron metabolism, all of which critically influence ferroptosis susceptibility. For instance, T98G cells are characterized by high MGMT expression and mutant p53 status, exhibit strong resistance to TMZ, yet demonstrate increased dependence on redox homeostasis, rendering them particularly vulnerable to ferroptosis induction via system xc^−^ inhibition [64]. In contrast, U87 cells display lower MGMT expression and relative TMZ sensitivity but exhibit more moderate ferroptotic responsiveness, likely reflecting differences in intracellular iron handling and oxidative buffering capacity [4,5]. The LN18 and LN229 models further illustrate this heterogeneity; LN18 cells exhibit enhanced resistance associated with oxidative stress tolerance, whereas LN229 cells show increased susceptibility to GPX4 inhibition and lipid peroxidation [71]. Notably, GSCs (e.g., MGG18, GSC#1) exhibit elevated NRF2-driven antioxidant defences and iron metabolic activity, conferring resistance to apoptosis while simultaneously creating a ferroptosis-prone state when redox balance is disrupted [62,64].

These observations indicate that ferroptosis sensitivity in GBM is not merely variable but is fundamentally dictated by the underlying metabolic and redox architecture of each model. Consequently, differences in therapeutic response across studies reflect intrinsic molecular heterogeneity rather than experimental inconsistency, underscoring the importance of model-specific interpretation in ferroptosis-based strategies.

### 3.3. Role of Ferroptosis Induction in GBM

Ferroptosis modulation consistently attenuated acquired and inherent TMZ resistance. Several small molecules induce ferroptosis and thereby affect GBM sensitivity to TMZ. For instance, Hacioglu et al. [65] demonstrated that capsaicin sensitized U87-R cells, reducing the TMZ IC_50_ from 912.7 µM to 522.6 µM. Similarly, Hacioglu and Tuncer [3] reported that boric acid triggered NCOA4-mediated ferritinophagy in A172-R cells, shifting the IC_50_ from 921.4 µM to 604.1 µM. Genetic modulation of iron metabolism showed robust effects in acquired resistance models. Lan et al. [66] reported that IRP1 overexpression reversed the NF-κB2-mediated iron export axis, significantly lowering the IC_50_ from 1673.0 µM [325.0 µg/mL] to 531.9 µM [103.3 µg/mL], representing a 3.1-fold increase in sensitivity. Yang et al. [71] demonstrated that pharmacological GPX4 inhibition with RSL3 enhanced cytotoxicity in LN18 cells, reducing IC_50_ values from 2.38 µM [2376 nM] to 0.45 µM [446 nM], a 5.3-fold reduction. In models of inherent resistance, de Souza et al. [64] identified “collateral sensitivity,” showing that NRF2-high T98G cells, while resistant to TMZ, were hypersensitive to Erastin, resulting in <40% cell viability following system xc^−^ blockade.

### 3.4. Nanoparticle-Mediated Resensitization of GBM to TMZ

Nanoparticle platforms demonstrated the greatest TMZ potentiation by facilitating targeted delivery and controlled release. Stephen et al. [67,68] developed redox-responsive, pH-triggered nanoparticles that restored near-total susceptibility in SF767 cells. These platforms shifted the LD_10_ from 640.0 µM to 15.7 µM (40.7-fold change) and 487.0 µM to 12.1 µM (40.2-fold change), respectively. In GSC models, Abu-Serie [62] reported that dual-enzyme iron oxide nanoparticles (DE-FeONPs) targeting ALDH1A1 reduced the IC_50_ in resistant MGG18-RR variants from 2497.0 µM [485 µg/mL] to 731.0 µM [142 µg/mL]. For MGMT-mediated resistance, Yoo et al. [72] utilized siMGMT-functionalized iron oxide nanoparticles to shift the LD_50_ in T98G cells from 8000 µM [8.0 mM] to 2800 µM [2.8 mM], effectively achieving a 65% increase in sensitivity by pairing DNA repair inhibition with nanoparticle-driven reactive oxygen species (ROS) induction.

### 3.5. In Vivo Therapeutic Response to TMZ and Survival Analysis

The therapeutic efficacy of these strategies was validated in orthotopic murine models. Su et al. [69] demonstrated that Roxadustat-mediated HIF-2α activation fuelled lipid ROS accumulation, extending median survival from 26 days in TMZ-monotherapy mice to 40 days in the combination cohort. Lan et al. [66] showed that IRP1 overexpression prolonged survival from 42 to 55 days. Alternative delivery routes were also explored to bypass biological barriers. Sukumar et al. [70] utilized intranasal delivery of gold-iron oxide nanoparticles (polyGIONs) loaded with miR-100/antimiR-21. This non-invasive strategy bypassed the BBB and reduced tumour volume by 42%, significantly increasing survival. Histological analysis by Buccarelli et al. [63] confirmed that quinacrine-induced death in GSCs was iron-dependent, as survival was reduced to 25% and the effect was significantly reversed by the iron chelator DFO and the ferroptosis inhibitor Ferrostatin-1.

### 3.6. Publication Bias and Heterogeneity

**Figure 2:**
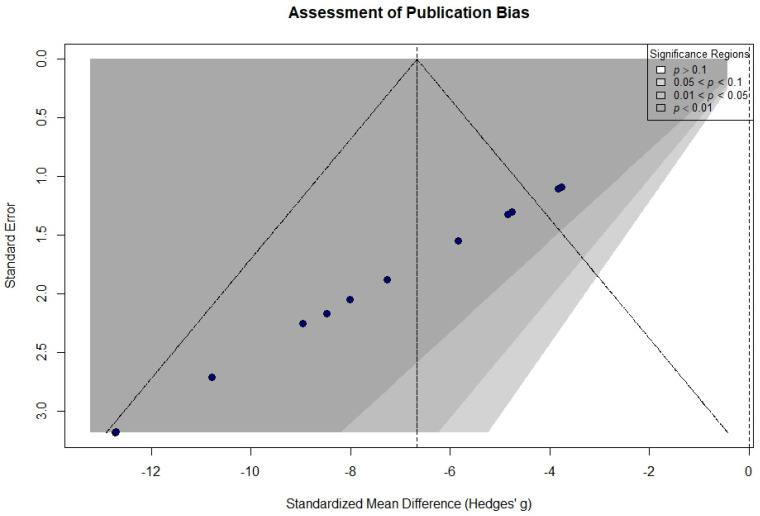
Contour-enhanced funnel plot for assessment of publication bias. Standardized Mean Difference (Hedges’ g) is plotted against the standard error to evaluate the distribution of study effects. The shaded regions represent different levels of statistical significance.

Heterogeneity was moderate across studies (I^2^ = 57.4%), primarily reflecting the distinction between acquired and inherent resistance mechanisms. Publication bias was evaluated using contour-enhanced funnel plots (Figure 2) and Egger’s linear regression test, which indicated asymmetry (t = −157.74, *p* < 0.0001). However, the majority of studies were conducted in high-significance regions (*p* < 0.01), suggesting that the asymmetry was driven by the superior therapeutic effect of nanoparticle-based delivery rather than by the suppression of negative results.

### 3.7. Risk of Bias and Quality Assessment

The overall risk of bias was found to be Low for reporting and baseline similarities across all studies (Table 5). Selection bias (including sequence generation) was Low for most in vivo studies, as this explicitly mentioned randomization. Detection bias was rated Low for Stephen et al. [68] and Sukumar et al. [70], as these studies utilized automated quantification and blinded analysis of neuroimaging and histological data. The performance bias domains were rated unclear due to the lack of reporting of specific laboratory housing protocols and caregiver blinding.

- Q1 (Selection Bias): Was the allocation sequence adequately generated and applied? (Evaluates if animals were truly randomized to groups).
- Q2 (Selection Bias): Were the groups’ baseline characteristics similar? (Evaluates age, weight, and starting tumour volume).
- Q3 (Selection Bias): Was the allocation adequately concealed? (Evaluates if researchers knew which animal was in which group at the start).
- Q4 (Performance Bias): Were the animals housed randomly during the experiment?
- Q5 (Performance Bias): Were the caregivers and/or investigators blinded from the knowledge of which intervention each animal received?
- Q6 (Detection Bias): Were animals selected at random for outcome assessment?
- Q7 (Detection Bias): Was the outcome assessor blinded? (e.g., were the MRI or histology slides read by someone unaware of the treatment group?).
- Q8 (Attrition Bias): Were all animals accounted for, and were incomplete outcome data addressed?
- Q9 (Reporting Bias): Are reports of the study free of selective outcome reporting? (Do the results match the stated methods?).
- Q10 (Other Bias): Was the study apparently free of other problems that could result in a high risk of bias?

### 3.8. Statistical Analysis

**Figure 3:**
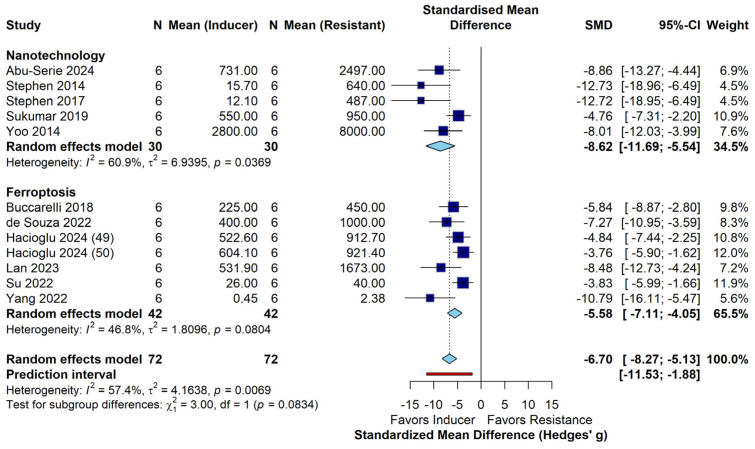
Meta-analysis of the sensitization effect of ferroptosis induction on TMZ-resistant GBM [3,62,63,64,65,66,67,68,69,70,71,72]. Standardized Mean Difference (SMD) and 95% Confidence Intervals (CIs) were calculated using a random-effects model (Hedges’ g) to account for variations in study design.

The overall pooled SMD of −6.70 [95% CI: −8.27, −5.13] suggested a strong therapeutic effect; the moderate heterogeneity (I^2^ = 57.4%) observed in this meta-analysis indicates a careful consideration of the findings. Studies on acquired resistance (e.g., Hacioglu, Lan) showed uniform IC50 shifts, probably because they involved defined genetic modifications or standardized induction of drug-resistant cell lines. Models of inherent resistance (e.g., Buccarelli, de Souza) showed greater variability, reflecting the basal molecular diversity of GSC lines and established cell lines such as T98G. The subgroup analysis as shown in Figure 3 that nanoparticle-mediated delivery systems achieved a higher magnitude of sensitization (pooled SMD: −8.62) than small-molecule interventions (pooled SMD: −5.58), although this difference did not reach statistical significance (*p* = 0.08). The significant asymmetry on Egger’s test (t = −157.74, *p* < 0.0001) was probably due to the magnitude effect rather than a publication bias.

## 4. Discussion

The findings of this systematic review and meta-analysis, characterized by an overall standardized mean difference (SMD) of −6.70 (95% CI: [−8.27, −5.13]; *p* < 0.0001), provide definitive preclinical evidence that the induction of ferroptosis and the implementation of nanoparticle-mediated delivery systems represent a transformative strategy for overcoming TMZ resistance in GBM. For decades, the clinical management of grade 4 gliomas has been stymied by a multifaceted resistance landscape, primarily driven by the DNA repair enzyme MGMT and defects in the p53-mediated apoptotic pathway. The traditional therapeutic paradigm, which relies on alkylation-induced apoptosis, frequently fails as tumour cells, particularly GSCs, leverage robust antioxidant defences and DNA damage response (DDR) mechanisms to survive. However, the synthesis of the 12 included studies suggests a fundamental shift in perspective: while GBM cells are adept at evading apoptosis, they remain highly vulnerable to iron-dependent lipid peroxidation, a process that can be strategically triggered to bypass established chemoresistance. However, these findings should be interpreted in the context of preclinical evidence, as all included studies were conducted in experimental models rather than clinical settings. The included studies also employed heterogeneous GBM systems, including established GBM cell lines, GBM stem-like cells, orthotopic xenografts, and diverse nanoparticle formulations, which may introduce biological variability and limit direct clinical translation.

A central mechanistic pillar identified in this review is the modulation of cellular iron homeostasis, specifically the restoration of the labile iron pool (LIP) to fuel the lethal Fenton reaction. Acquired TMZ resistance is frequently associated with the loss of Iron Regulatory Protein 1 (IRP1), which Lan et al. [66] identified as a critical modulator of chemosensitivity. The down-regulation of IRP1 activates a non-canonical NF-κB2 signalling pathway that upregulates iron export mechanisms, effectively shielding the cell from oxidative damage by maintaining low intracellular iron levels. By reversing this axis through IRP1 overexpression, intracellular iron is sequestered, leading to a significant reduction in IC_50_ from 1673.0 µM [325.0 µg/mL] to 531.9 µM [103.3 µg/mL], representing a 3.1-fold increase in sensitivity. This iron-trapping strategy is complemented by the induction of NCOA4-mediated ferritinophagy, as demonstrated by Hacioglu and Tuncer [3]. The use of boric acid to trigger the selective autophagic degradation of ferritin complexes liberates stored iron into the cytosol, providing the necessary substrate for reactive oxygen species (ROS) accumulation. This convergence of trapping iron via the IRP1 axis and liberating it via the NCOA4 axis creates an intracellular environment “primed” for ferroptosis, rendering even the most refractory A172-R and U87-R cells susceptible to combination therapy.

Beyond iron sequestration, the success of ferroptosis-inducing strategies relies on the simultaneous disruption of the cell’s antioxidant defence machinery, specifically the system xc^−^/GSH/GPX4 axis. The direct inhibition of glutathione peroxidase 4 (GPX4) by small molecules such as RSL3, as explored by Yang et al. [71], demonstrated a 5.3-fold increase in sensitivity, shifting the IC_50_ from 2.38 µM [2376 nM] to 0.45 µM [446 nM]. This pharmacological intervention forces the accumulation of lethal lipid peroxides, leading to catastrophic membrane rupture. Perhaps the most intriguing biological finding in this synthesis is the phenomenon of “collateral sensitivity,” described by de Souza et al. [64]. Conventionally, high NRF2 expression is viewed as a survival advantage that protects GBM cells from oxidative stress and chemotherapy. However, this study reveals that NRF2-high, TMZ-resistant T98G cells become hypersensitized to ferroptosis upon the blockage of the system xc^−^/ABCC1 axis. In this paradigm, the very mechanisms used to maintain redox homeostasis create a fatal dependency; once glutathione (GSH) efflux is triggered, the cell’s defences collapse more rapidly than in sensitive variants. This suggests that the most chemoresistant GBM subtypes, those overexpressing NRF2 and MGMT, may paradoxically be the ideal candidates for pro-ferroptotic interventions.

The clinical translation of these biological insights, however, depends on overcoming the formidable BBB and minimizing systemic off-target toxicities.

However, it is important to note that not all nanoparticle-induced cytotoxicity can be attributed exclusively to ferroptosis. Certain nanomaterials may induce cell death through nonspecific mechanisms, including cellular accumulation, membrane disruption, or generalized oxidative stress independent of canonical ferroptotic pathways. Therefore, careful validation using ferroptosis-specific markers (e.g., lipid peroxidation, GPX4 inhibition, ferrostatin-1 rescue) is essential to distinguish true ferroptotic effects from nonspecific nanoparticle toxicity. This distinction is critical, as attributing nanoparticle-mediated cytotoxicity solely to ferroptosis without mechanistic validation may lead to overinterpretation of therapeutic efficacy.

Notably, nanoparticles can induce ferroptosis beyond their conventional role as passive delivery vehicles. Iron-based nanomaterials, such as Fe_3_O_4_ and FeONPs, actively participate in redox cycling by catalyzing Fenton reactions, thereby generating hydroxyl radicals and amplifying lipid peroxidation within tumour cells [56,62]. This intrinsic catalytic activity promotes the accumulation of ROS and directly drives ferroptotic cell death.

Furthermore, certain nanoplatforms disrupt intracellular antioxidant defences by depleting GSH or impairing GPX4 activity, thereby lowering the threshold for ferroptosis induction [18]. Together, these findings redefine nanoparticles as active biochemical modulators rather than inert carriers, integrating targeted delivery with catalytic induction of ferroptotic signalling. This dual functionality represents a significant conceptual advance in nanotherapeutic design, enhancing both the specificity and efficacy of strategies to overcome TMZ resistance in GBM. However, it is important to note that not all nanoparticle-induced cytotoxicity can be attributed exclusively to ferroptosis. Certain nanomaterials may induce cell death through nonspecific mechanisms, including cellular accumulation, membrane disruption, or generalized oxidative stress independent of canonical ferroptotic pathways. Therefore, careful validation using ferroptosis-specific markers (e.g., lipid peroxidation, GPX4 inhibition, ferrostatin-1 rescue) is essential to distinguish true ferroptotic effects from nonspecific nanoparticle toxicity. This distinction is critical, as attributing nanoparticle-mediated cytotoxicity solely to ferroptosis without mechanistic validation may lead to overinterpretation of therapeutic efficacy.

This review’s subgroup analysis provides a critical finding: nanoparticle-mediated delivery systems achieved a significantly greater level of sensitization than small-molecule inhibitors (*p* = 0.0834). The clinical failure of free O6-benzylguanine (BG) in previous human trials was largely due to dose-limiting myelosuppression and poor intracranial accumulation. The nanoparticle platforms developed by Stephen et al. [67,68] and Yoo et al. [72] address these hurdles through precision engineering. By utilizing pH-sensitive hydrazone linkages and redox-responsive polymer shells, these nanoparticles ensure that therapeutic payloads (such as siMGMT or BG) are released exclusively within the acidic, reductive tumour microenvironment. This spatial and temporal control achieved a near-total restoration of susceptibility in SF767 models, shifting the LD_10_ from 640.0 µM to 15.7 µM, an unprecedented 40.7-fold change. This targeted approach not only maximizes the drug’s potency at the tumour site but also protects healthy bone marrow from alkylation-induced damage, effectively widening the therapeutic window for TMZ therapy.

Further advancing the translational landscape is the successful application of alternative delivery routes to bypass the BBB. Sukumar et al. [70] provided a clinical blueprint for non-invasive intranasal administration, using gold-iron oxide nanoparticles (polyGIONs) to deliver therapeutic miRNAs (miR-100 and antimiR-21). This route achieves direct brain accumulation via the olfactory and trigeminal nerves, resulting in a 42% reduction in tumour volume. When combined with the MRI-targeted iron delivery as reported by Yoo et al. [72], these strategies offer a feasible path to treating deep-seated or inoperable GBM lesions that remain refractory to standard-of-care. Furthermore, the integration of iron oxide cores into these delivery systems meets the “theranostic” requirement, enabling clinicians to monitor nanoparticle trafficking and tumour volume changes in real time via magnetic resonance imaging. This synthesis of delivery and diagnostics is essential for the personalized management of GBM, where tumour heterogeneity often dictates varying responses to therapy.

The efficacy of ferroptosis induction is particularly pronounced when targeting the GSC niche, which is the primary driver of tumour recurrence. GSCs are notoriously resistant to apoptosis and often utilize autophagy as a constitutive cytoprotective mechanism. Buccarelli et al. [63] and Abu-Serie [62] demonstrated that disrupting this niche through autophagy blockade with Quinacrine or ALDH1A1 suppression with dual-enzyme iron oxide nanoparticles (DE-FeONPs) triggers iron-dependent death. Because GSCs have a higher iron requirement to sustain self-renewal, they are naturally “primed” for the Fenton reaction. Abu-Serie reported that DE-FeONPs reduced the TMZ IC_50_ in resistant MGG18-RR variants by 70.7%, effectively inhibiting self-renewal and radio-resistance. This highlights that ferroptosis induction is not merely a strategy to kill bulk tumour cells but a potent tool to “uproot” the resilient stem cell populations that currently limit the median survival of GBM patients to 15–18 months.

The role of the hypoxic microenvironment also emerges as a nuanced factor in this review. While hypoxia is traditionally associated with therapy resistance, Su et al. [69] demonstrated that stabilizing the hypoxia-inducible factor (HIF) pathway with Roxadustat can fuel lipid ROS accumulation via the HIF-2α/PLIN2 axis. This finding suggests that the hypoxic core of a GBM tumour, which is ordinarily the most radiotherapy-resistant region, could be specifically sensitized by modulating lipid-regulatory genes. The ability of Roxadustat to extend median survival from 26 to 40 days in chemoresistant murine models underscores the potential for repurposing existing prolyl hydroxylase (PHD) inhibitors to hypersensitize tumours to oxidative death. tumour Similarly, the TMZ-ferroptosis relationship may lead to metabolic reprogramming that can turn the tumour’s microenvironment into a lethal consequence.

**Figure 4:**
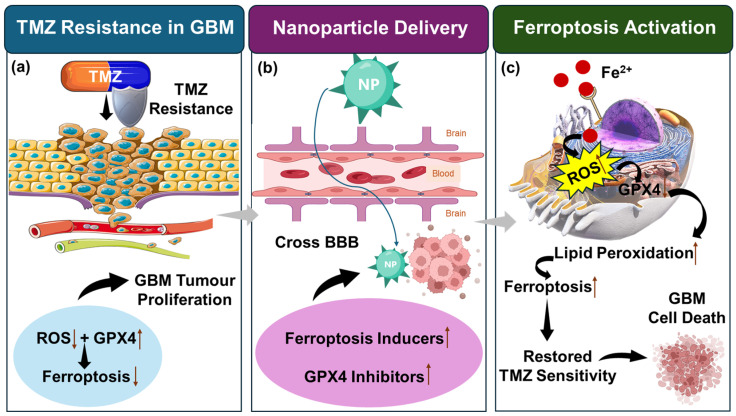
Schematic illustration of NP-mediated ferroptosis activation to overcome TMZ resistance in GBM. (**a**) In TMZ-resistant GBM, tumour cells evade therapy and continue to proliferate while ferroptosis signalling remains suppressed. Wherein, ROS accumulation and elevated antioxidant defence mechanisms, including GPX4 activity, contribute to resistance and decreased ferroptotic cell death. (**b**) NP-based delivery systems enable efficient transport of therapeutic agents across the BBB. These nanocarriers deliver ferroptosis-inducing compounds and GPX4 inhibitors directly to tumour cells within the TME. (**c**) Ferroptosis is activated by increased intracellular iron-mediated ROS generation and GPX4 inhibition, leading to lipid peroxidation and ferroptotic tumour cell death. This mechanism ultimately restores TMZ sensitivity and suppresses GBM tumour growth. Created using Microsoft PowerPoint with icons adapted from BioRender (created in BioRender https://BioRender.com, accessed on 16 March 2026), Bioicons, and Servier Medical Art. Footnote: Upward arrow ↑ indicates upregulation, downward arrow ↓ indicates downregulation.

Recent evidence highlights an emerging crosstalk between ferroptosis and the immune microenvironment in GBM [73,74]. Ferroptotic tumour cells generally release lipid peroxidation products and damage-associated molecular patterns (DAMPs), which stimulate immune activation within the tumour microenvironment. Notably, CD8^+^ T cells have been shown to promote tumour ferroptosis through interferon-γ (IFN-γ)-mediated suppression of the system xc^−^ antioxidant pathway, thereby enhancing lipid peroxidation and ferroptotic cell death. In parallel, ferroptosis can influence tumour-associated macrophages by promoting a pro-inflammatory phenotype that may counteract the highly immunosuppressive microenvironment characteristic of GBM [73,75,76]. These findings suggest that ferroptosis-inducing therapies may exert dual anticancer effects by directly killing tumour cells and simultaneously reshaping immune responses. When combined with nanoparticle-based delivery systems capable of crossing the BBB and selectively targeting tumour tissues, ferroptosis induction may therefore represent a particularly effective strategy to overcome TMZ resistance while also modulating immune components of the GBM microenvironment. The mechanisms linking TMZ resistance, ferroptosis signalling, and nanoparticle-mediated delivery strategies are shown in Figure 4.

As TMZ remains the current standard chemotherapeutic agent for GBM, this review primarily focuses on mechanisms underlying TMZ resistance. However, emerging evidence suggests that ferroptosis constitutes a broader, convergent vulnerability underlying resistance to multiple therapeutic modalities. In GBM, resistance to radiotherapy and experimental treatment strategies is frequently associated with enhanced antioxidant defences, dysregulated iron metabolism, and lipid remodelling, all of which are key regulators of ferroptosis susceptibility [14,15,16,18]. Importantly, recent studies demonstrate that alternative chemotherapeutic agents, such as cisplatin, can directly induce ferroptosis by increasing lipid peroxidation and depleting antioxidant systems, including GSH and GPX4, thereby promoting oxidative cell death pathways [77]. However, the clinical application of cisplatin in GBM is limited by its poor BBB permeability, which restricts its effective delivery to tumour tissue [78].

Taken together, these findings position ferroptosis not as a TMZ-specific mechanism but as a shared metabolic liability across resistant cancer states. Targeting this vulnerability, particularly in combination with strategies that enhance drug delivery across the BBB, may expand therapeutic options and improve outcomes beyond TMZ-centred regimens in GBM.

### 4.1. Strengths and Limitations

This systematic review is, to our knowledge, the first to comprehensively synthesize and quantitatively analyze preclinical evidence on ferroptosis and nanoparticle-based strategies to overcome TMZ resistance in GBM. By integrating diverse molecular regulators (e.g., IRF2, FHOD1, NCOA4, IRP1, HIF-2α, and MGMT) and various ferroptosis inducers (capsaicin, boric acid, RSL3, DE-FeONPs), this review highlights convergent mechanistic pathways that govern chemoresistance. The quantitative reduction in TMZ IC_50 across independent models provides robust functional evidence of ferroptosis-mediated chemosensitization. Furthermore, stratifying studies into subgroups allows the objective conclusion that, while all ferroptotic inducers are beneficial, nanoparticle-mediated delivery provides a statistically superior level of sensitization (*p* = 0.0834 for subgroup differences). This review also emphasizes GSCs and resistant variants, clinically relevant populations that drive recurrence and therapeutic failure, thereby strengthening the clinical applicability of these findings. Several limitations must be acknowledged. First, all included studies were preclinical; thus, the findings may not fully translate to human patients due to interspecies differences, marked tumour heterogeneity, and the complex GBM microenvironment. Moreover, several studies relied on simplified in vitro cellular models, which cannot fully replicate the spatial heterogeneity, immune interactions, and metabolic gradients present in the in vivo tumour environment. Second, considerable methodological heterogeneity was observed across studies, particularly regarding the “Acidosis Gap” identified by Buccarelli et al. (2018) [63], in which extreme extracellular low pH (6.5) can inhibit the efficacy of certain agents, posing a challenge for drugs not specifically engineered for acidic environments. Third, while reductions in IC_50 values provide a measurable indicator of restored sensitivity, they may not fully capture long-term clinical outcomes such as delayed recurrence or adaptive resistance. Fourth, reliance on immunodeficient mouse models in most studies may lead to an underestimation of ferroptosis’s immunogenic potential, as T-cell recruitment and the full “immunogenic cell death” cascade cannot be modelled without a competent immune system. Fifth, differences in glioma classification across historical studies represent an additional source of heterogeneity. Several investigations were conducted before the implementation of the WHO 2021 classification of central nervous system tumours, which distinguishes glioblastoma (astrocytoma, IDH-wildtype, CNS WHO grade 4) from astrocytoma, IDH-mutant, CNS WHO grade 4. Because these entities exhibit distinct molecular characteristics and therapeutic responses, their differential representation across preclinical models may influence the interpretation of ferroptosis-based sensitization strategies. Moreover, the long-term neuro-accumulation and metabolic clearance of metallic nanoparticle cores remain understudied, requiring further longitudinal safety assessments before clinical translation.

### 4.2. Future Directions

The translational transition of ferroptosis-inducing nanoparticles from preclinical models to the clinical management of GBM requires several critical advancements. First, the investigation of a “triple-synergy” therapeutic regimen combining radiotherapy, TMZ, and targeted ferroptotic stimuli is warranted. Since ionizing radiation facilitates the Fenton reaction by generating hydroxyl radicals, combining it with iron-trapping strategies (e.g., IRP1 overexpression or NCOA4-mediated ferritinophagy) could lower the dose requirements for both radiation and chemotherapy, thereby reducing systemic side effects. Second, identifying predictive biomarkers is essential for patient stratification. Clinicians could utilize basal NRF2 activity, MGMT methylation status, and ALDH1A1 expression levels to identify “poor responders” who are biologically predisposed to collateral sensitivity. This precision oncology framework would enable the deployment of potent oxidative-lethal stimuli in patients most likely to benefit from the dismantling of the system xc^−^/ABCC1 axis. Technological refinement must also focus on the long-term safety and multi-targeting capabilities of next-generation nanoparticles. Future platforms should aim to combine T7 peptide-mediated BBB penetration with ligands that specifically target the hypoxic, HIF-2α-rich tumour core to ensure deeper parenchymal accumulation. Longitudinal studies are required to characterize the metabolic fate and neuro-clearance of metallic cores, such as the gold and iron-oxide architectures analyzed in this review, to preclude chronic neuro-accumulation. Furthermore, the clinical adoption of non-invasive administration routes, particularly the intranasal pathway, will require the development of medical-grade nebulization devices capable of delivering precise, reproducible doses to the olfactory and trigeminal nerve branches. Finally, future research must shift toward immunocompetent, syngeneic murine models to fully elucidate the interaction between ferroptosis-induced immunogenic cell death and the immunosuppressive GBM microenvironment. Understanding how iron-dependent membrane rupture recruits T cells and modulates the tumour microenvironment will be key to transforming these preclinical oxidative triggers into a durable clinical solution to prevent GBM recurrence.

## 5. Conclusions

In conclusion, this systematic review and meta-analysis demonstrate that integrating ferroptosis-inducing strategies with nanoparticle-based delivery represents a superior approach to overcoming TMZ resistance in GBM. By simultaneously targeting the cell’s iron regulatory machinery (IRP1, NCOA4), antioxidant defences (GPX4, NRF2), and DNA repair systems (MGMT), these multimodal interventions achieve a near-total restoration of chemosensitivity in preclinical models. The numerical superiority of nanoparticle-based platforms in our subgroup analysis (pooled SMD: −8.62 vs. −5.58; *p* = 0.08) highlights that precision delivery is likely a prerequisite for achieving clinical relevance. Future clinical directions must prioritize the translation of these targeted nanoparticle platforms, particularly those that utilize non-invasive delivery routes, such as the intranasal route, to deliver lethal oxidative stimuli directly to the chemoresistant tumour niche. By shifting the therapeutic focus from alkylation-induced apoptosis to iron-dependent lipid peroxidation, this multimodal strategy provides a potent therapeutic window to eradicate both the differentiated tumour bulk and the resilient GBM stem cell populations that drive disease recurrence and mortality.

## Figures and Tables

**Table 1:** Details of the search strategy.

Database	Search Details
COCHRANE	0 Cochrane Reviews matching iron glioblastoma temozolomide in Title, Abstract, and Keywords
PubMed	((“iron”[MeSH Terms] OR “iron”[All Fields]) AND (“glioblastoma”[MeSH Terms] OR “glioblastoma”[All Fields] OR “glioblastomas”[All Fields]) AND (“temozolomide”[MeSH Terms] OR “temozolomide”[All Fields] OR “temozolomide s”[All Fields])) AND (1000/1/1:2025/4/3[pdat])
ScienceDirect	Title, abstract, keywords: iron glioblastoma temozolomide
SCOPUS	TITLE-ABS-KEY (iron AND glioblastoma AND temozolomide)

**Table 2:** Details of PICO question.

Component	Definition
Population	Preclinical glioblastoma models (in vitro cell lines, organoids; in vivo animal/xenograft/PDX) with demonstrated or induced temozolomide (TMZ) resistance (e.g., MGMT ↑ lines, chronically TMZ-exposed lines, resistant PDX).
Intervention	Strategies that induce or modulate ferroptosis (e.g., erastin, RSL3, GPX4 inhibition, iron metabolism targeting, ferritinophagy) and/or nanoparticle-based delivery systems (liposomes, polymer/magnetic NPs, exosomes, micelles) used alone or with TMZ.
Comparator	TMZ-sensitive models, vehicle/untreated controls, or alternative non-ferroptosis/non-nanotech interventions (e.g., free TMZ vs. nano-TMZ; TMZ alone vs. TMZ + ferroptosis inducer).
Outcomes (Primary)	Reduction in TMZ resistance: improved TMZ sensitivity (IC_50_ ↓), tumour shrinkage/volume ↓, survival benefit in vivo, synergism indices with TMZ.
Outcomes (Secondary)	Mechanistic markers of ferroptosis and resistance: lipid ROS/MDA ↑, GSH ↓, GPX4 ↓, SLC7A11/xCT ↓, IRP1/LCN2/FPN1 axis changes, MGMT modulation, delivery/biodistribution metrics for nanotech.

**Table 3:** Characteristics of excluded studies.

Study Author (Year)	Reason for Exclusion	Exclusion Category
Alexiou, 2016 [23]	Only tests iron chelator (deferiprone) in general glioma cells; no ferroptosis or nanotech strategy.	Inappropriate intervention
Calzolari, 2010 [24]	Observational study on transferrin receptor expression; no TMZ resistance intervention.	No therapeutic outcomes
Chen, 2023 [25]	miR-10b targeting; not ferroptosis or nanoparticle focused on TMZ resistance	Inappropriate intervention
Cushing, 2021 [26]	Clinical biomarker study; not preclinical or intervention-based.	Clinical trial/Review
El Husseini, 2020 [27]	Case report; clinical, not preclinical.	Clinical trial/Review
Fontanilles, 2019 [30]	Clinical platelet monitoring; no preclinical intervention.	Clinical trial/Review
Fontanilles, 2020 [28]	Clinical comparison of TMZ formulations; no ferroptosis/nanotech mechanism.	Clinical trial/Review
Fontanilles, 2020 [31]	Clinical cfDNA/TERT mutation monitoring; not intervention-based.	Clinical trial/Review
Fontanilles, 2020 [32]	Clinical EGFR detection study; no preclinical TMZ resistance model.	Clinical trial/Review
Fontanilles, 2024 [29]	Multi-omics clinical study; not preclinical, no TMZ resistance intervention.	Clinical trial/Review
Huang, 2017 [33]	Nanotherapeutics via stem cells; lack of TMZ-resistant context or ferroptosis analysis	No TMZ-resistance model
Kun, 2016 [34]	Pituitary adenoma model, not GBM; mechanism not ferroptosis/nanotech.	Inappropriate intervention
Kwon, 2019 [35]	TMZ/ICG-iron oxide NPs chemo-phototherapy without TMZ-resistant model	No TMZ-resistance model
Lee, 2021 [37]	Immunomodulation target (IL-19); no TMZ/ferroptosis focus	Inappropriate intervention
Lee, 2025 [36]	Diagnostic PD-L1 imaging in TMZ-resistant GBM; not a therapeutic overcoming resistance	No therapeutic outcomes
Li, 2022 [38]	Ferritinophagy/ferroptosis mechanistic work; no TMZ-resistant model	No TMZ-resistance model
Liu, 2020 [39]	Focus on hypoxia and FTL; no ferroptosis/nanotech strategy in TMZ-resistant GBM.	No therapeutic outcomes
Lozano-Gonzalez, 2018 [40]	Novel compound study; general anticancer, not TMZ resistance-focused.	No TMZ-resistance model
Mohanty, 2017 [41]	Theranostic MMP-14 targeting; not TMZ/ferroptosis in resistance setting	No TMZ-resistance model
Mouawad, 2023 [42]	Sensitizes to topo II inhibitors; not TMZ	Inappropriate intervention
Nozhat, 2024 [43]	TMZ-loaded Fe_3_O_4_@SiO_2_ NPs in vitro; lacks TMZ-resistant model/sensitization endpoint	No TMZ-resistance model
Petronek, 2024 [44]	Clinical phase 2 trial; biomarker study only, no preclinical intervention.	Clinical trial/Review
Petronek, 2024 [45]	Clinical imaging study; not preclinical or mechanistic.	Clinical trial/Review
Prabhu, 2017 [46]	Nestin-targeting TMZ nanocomposite; no explicit TMZ-resistant model	No TMZ-resistance model
Rivera, 2025 [47]	Magnetic hyperthermia with chemoradiation; not TMZ-specific or ferroptosis-based	Inappropriate intervention
Senturk, 2022 [48]	Formulation feasibility/challenges; not therapeutic efficacy vs. resistance	No therapeutic outcomes
Seyfoori, 2025 [49]	Tumouroid-on-a-plate model; general platform development, not TMZ resistance-specific.	No TMZ-resistance model
Song, 2021 [50]	TMZ-induced ferroptosis mechanism; not overcoming TMZ resistance	No TMZ-resistance model
Stefan, 2025 [51]	Clinical phase I trial; no preclinical focus.	Clinical trial/Review
Su, 2023 [52]	CYBB/Nrf2/SOD2 axis; chemo/ferroptosis resistance broadly; TMZ-specific resistant model unclear	No TMZ-resistance model
Tang, 2024 [53]	Biomimetic NPs for chemo-radiation; TMZ-resistant context not addressed	No TMZ-resistance model
Wang, 2021 [54]	siRNA NPs vs. drug-resistant gene; TMZ specificity unclear	No TMZ-resistance model
Wang, 2023 [55]	General molecular study on TRIM7; no nanotech or ferroptosis intervention with TMZ.	No therapeutic outcomes
Wang, 2023 [56]	Ferroptosis induction platform; no TMZ-resistant model	No TMZ-resistance model
Williams, 2024 [57]	Prosurvival pathway (acidic pH); not TMZ or ferroptosis-targeted resistance	No therapeutic outcomes
Xu, 2022 [58]	Ferroptosis via miR-147a; TMZ-resistant model not addressed	No therapeutic outcomes
Yang, 2004 [59]	Early in vitro study on cobalt-induced chemoresistance; unrelated to ferroptosis/nanotech.	Inappropriate intervention
Yin, 2024 [60]	Clinical or translational; not preclinical ferroptosis/nanotech.	Clinical trial/Review
Zhu, 2019 [61]	Anti-GBM agent study; not specifically addressing TMZ resistance via ferroptosis/nanotech.	No TMZ-resistance model

**Table 4:** Comparative Analysis of Ferroptosis Induction Strategies in TMZ-Resistant GBM.

Study Authors (Year)	Country	Study Type	Cell Line	Animal Model	Route	IC_50_/LD_10_ Parental	IC_50_/LD_10_ Resistant	IC_50_/LD_10_ with Inducer	Fold-Change (↓)	% Sens. Increase	Mechanism/Intervention	Sensitivity/Resistance Outcomes
Abu-Serie, 2024 [62]	Egypt/USA	In vitro	Human GSCs	None	NA	1349.0 µM (MGG18)	2497.0 µM	731.0 µM	3.4×	70.7%	DE-FeONPs (Nanoparticle): Direct iron delivery via nanoparticles inducing Fenton-reaction-mediated ferroptosis.	Acquired Resistance. DE-FeONPs inhibited GSC self-renewal and re-sensitized cells to TMZ and radiation.
Buccarelli, 2018 [63]	Italy	In vitro + In vivo	GSC#1, #163	Orthotopic	Intracranial	NA	NA (Inherent resistance)	↓ Survival to ~25% ^1^	NA	~50%	Quinacrine (Ferroptosis): Inhibition of autophagy, leading to iron-dependent ferroptotic cell death.	Inherent Resistance. In vitro, DFO/Fer-1 rescued cells, confirming ferroptosis. The in vivo effect was not significant.
de Souza, 2022 [64]	Brazil	In vitro	T98G, U251	None	NA	NA (Sensitive line used as comparator)	NA (Inherent resistance)	<40% Viability ^2^	NA	High	Erastin/RSL3 (Ferroptosis): Targeting NRF2/ABCC1 axis to enhance GSH efflux via system xc^−^ inhibition.	Inherent Resistance. Demonstrated “collateral sensitivity” where high NRF2 confers TMZ resistance but hypersensitizes cells to ferroptosis.
Hacioglu, 2024 [65]	Turkey	In vitro	U87, U251	None	NA	337.2 µM (U87)	912.7 µM	522.6 µM	1.7×	42.7%	Capsaicin (Ferroptosis): Downregulation of FHOD1/IRF2 signalling, leading to decreased GPX4/GSH.	Acquired Resistance. Restored ferroptotic susceptibility, induced cell cycle arrest, and reduced migration.
Hacioglu, 2024 [3]	Turkey	In vitro	A172, T98G	None	NA	362.8 µM (A172)	921.4 µM	604.1 µM	1.5×	34.4%	Boric Acid (Ferroptosis): Induction of ferritinophagy via upregulation of NCOA4/IRP2 pathway.	Acquired Resistance. Restored ferroptotic susceptibility by modulating cellular iron metabolism.
Lan, 2023 [66]	China	In vitro + In vivo	U87, U251	Orthotopic	IC/IP	595.3 µM (U87)	1673.0 µM	531.9 µM	3.1×	68.2%	IRP1 Overexpression (Ferroptosis): Reversal of IRP1 loss, which blocks NF-κB2 activation and subsequent iron export.	Acquired Resistance. In vivo, IRP1 overexpression significantly prolonged survival (55 vs. 42 days) in TMZ-treated mice.
Stephen, 2014 [68]	USA	In vitro + In vivo	SF767, GBM6	Orthotopic	IC (CED)	NA	640.0 µM ^3^	15.7 µM ^3^	40.7×	97.5%	BG-NP (Nanoparticle): Redox-responsive nanoparticle delivery of MGMT inhibitor (BG).	Inherent Resistance. Achieved a 3-fold increase in median survival and mitigated myelosuppression of free BG.
Stephen, 2017 [67]	USA	In vitro	SF767	None	NA	NA	487.0 µM ^3^	12.1 µM ^3^	40.2×	97.5%	BGS-NP (Nanoparticle): pH-triggered nanoparticle release of MGMT inhibitor (BGS analogue).	Inherent Resistance. High drug loading (33.4 wt%) and pH-specific release potentiated TMZ-induced apoptosis.
Su, 2022 [69]	China	In vitro + In vivo	GL261, U87	Orthotopic	IP	NA	N/A (Inherent resistance)	↑ Survival ^4^	1.5×	Significant	Roxadustat (Ferroptosis): Upregulation of HIF-2α, leading to increased lipid peroxidation.	Inherent Resistance. Roxadustat treatment alone prolonged survival more than TMZ, confirming efficacy in chemoresistant model.
Sukumar, 2019 [70]	USA	In vitro + In vivo	U87-MG	Orthotopic	Intranasal	950.0 µM	NA (Presensitization)	~42% Vol. ↓ ^5^	NA	42%	miRNA-NP (Nanoparticle): Intranasal delivery of NPs with miR-100/antimiR-21 to upregulate p53/PTEN pathways.	Presensitization. Intranasal route successfully bypassed the BBB, suppressed tumour growth, and increased survival.
Yang, 2022 [71]	China	In vitro + In vivo	LN18, LN229	Orthotopic	SC/IC	2.38 µM (LN18)	NA (Basal resistance)	0.45 µM	5.3×	81.2%	RSL3 (Ferroptosis): Direct inhibition of GPX4, leading to increased lipid peroxidation.	Inherent Resistance. Combination therapy prolonged survival and was effective in both IDH1-mutant and WT contexts.
Yoo, 2014 [72]	USA	In vitro + In vivo	T98G, U87	Orthotopic	Intratumoural	6.9 µM ^3^	8000 µM ^3^	↓ Survival to ~35% ^6^	NA	~65%	siMGMT-NP (Nanoparticle): Nanoparticle-mediated silencing of the MGMT DNA repair gene.	Inherent Resistance. MRI confirmed targeted NP delivery. The targeting agent (CTX) also contributed to cytotoxicity.

Footnotes: ^1^ Outcome represents the approximate reduction in surviving cell fraction at a fixed TMZ dose, not an IC_50_ value; ^2^ Outcome represents the remaining cell viability after treatment with 20 µM Erastin, demonstrating collateral sensitivity in a TMZ-resistant line; ^3^ Data represents LD_10_ (dose to reduce survival to 10%) values, not IC_50_; ^4^ Fold-change calculated from the ratio of median survival days in the intervention group vs. the TMZ-only group (40 d vs. 26 d); ^5^ Percentage refers specifically to in vivo tumour volume reduction compared to a non-targeted nanoparticle control; ^6^ Outcome represents the relative survival of TMZ-resistant tumours following siMGMT-NP pre-treatment and subsequent exposure to a fixed TMZ dose; Upward arrow ↑ indicates upregulation, downward arrow ↓ indicates downregulation. Abbreviations: IC: Intracranial; IP: Intraperitoneal; SC: Subcutaneous; CED: Convection Enhanced Delivery; GSCs: Glioblastoma Stem Cells; BBB: Blood–Brain Barrier; NA: Not Available/Not Applicable. The systematic review included 12 preclinical studies utilizing diverse GBM models, including established cell lines (U87, U251, T98G, LN18), patient-derived xenografts (GBM6), and glioblastoma stem-like cells (GSCs). Meta-analysis of these interventions demonstrated that inducing ferroptosis and using nanoparticle-mediated delivery systems significantly restored susceptibility to TMZ (Overall SMD: −6.70; 95% CI: [−8.27, −5.13]; *p* < 0.0001). Subgroup analysis revealed a strong numerical trend suggesting that nanoparticle-based platforms achieve greater sensitization magnitude (pooled SMD: −8.62) than small-molecule inhibitors (pooled SMD: −5.58), although the difference did not reach traditional statistical significance (*p* = 0.0834).

**Table 5:** Risk of Bias Assessment using SYRCLE’s Tool.

Study Author (Year)	Q1	Q2	Q3	Q4	Q5	Q6	Q7	Q8	Q9	Q10
Abu-Serie, 2024 [62]	Unclear	Low	Unclear	Unclear	Unclear	Unclear	Unclear	Low	Low	Low
Buccarelli, 2018 [63]	Low	Low	Unclear	Unclear	Unclear	Unclear	Unclear	Low	Low	Low
de Souza, 2022 [64]	Not Applicable	Low	Not Applicable	Not Applicable	Unclear	Unclear	Unclear	Low	Low	Low
Hacioglu, 2024 [65]	Not Applicable	Low	Not Applicable	Not Applicable	Unclear	Unclear	Unclear	Low	Low	Low
Hacioglu, 2024 [3]	Not Applicable	Low	Not Applicable	Not Applicable	Unclear	Unclear	Unclear	Low	Low	Low
Lan, 2023 [66]	Low	Low	Unclear	Unclear	Unclear	Unclear	Unclear	Low	Low	Low
Stephen, 2017 [67]	Low	Low	Unclear	Unclear	Unclear	Unclear	Low	Low	Low	Low
Stephen, 2014 [68]	Not Applicable	Low	Not Applicable	Not Applicable	Unclear	Unclear	Unclear	Low	Low	Low
Su, 2022 [69]	Low	Low	Unclear	Unclear	Unclear	Unclear	Unclear	Low	Low	Low
Sukumar, 2019 [70]	Low	Low	Unclear	Unclear	Unclear	Unclear	Low	Low	Low	Low
Yang, 2022 [71]	Low	Low	Unclear	Unclear	Unclear	Unclear	Unclear	Low	Low	Low
Yoo, 2014 [72]	Unclear	Low	Unclear	Unclear	Unclear	Unclear	Unclear	Low	Low	Low

## Data Availability

The data presented in this study are available on request from the corresponding author.

## Abbreviations

The following abbreviations are used in this manuscript:

GBM	Glioblastoma
TMZ	Temozolomide
ROS	Reactive Oxygen Species
GSH	Glutathione
GPX4	Glutathione Peroxidase 4
NRF2	Nuclear Factor Erythroid 2-Related Factor 2
MGMT	O6-Methylguanine-DNA Methyltransferase
BBB	Blood–Brain Barrier
GSCs	Glioblastoma Stem Cells
NPs	Nanoparticles
SMD	Standardized Mean Difference
IC_50_	Half Maximal Inhibitory Concentration
HIF	Hypoxia-Inducible Factor
LIP	Labile Iron Pool
DDR	DNA Damage Response
PRISMA	Preferred Reporting Items for Systematic Reviews and Meta-Analyses
PROSPERO	International Prospective Register of Systematic Reviews
OS	Overall Survival
WHO	World Health Organization
IDH	Isocitrate Dehydrogenase
CNS	Central Nervous System
PDX	Patient-Derived Xenograft
MDA	Malondialdehyde
xCT (SLC7A11)	Cystine/Glutamate Antiporter
IRP1	Iron Regulatory Protein 1
NF-κB	Nuclear Factor Kappa B
ABCC1	ATP-Binding Cassette Subfamily C Member 1
ALDH1A1	Aldehyde Dehydrogenase 1 Family Member A1
miRNA	MicroRNA
IFN-γ	Interferon Gamma
MRI	Magnetic Resonance Imaging
